# The actin cortex at a glance

**DOI:** 10.1242/jcs.186254

**Published:** 2018-07-19

**Authors:** Priyamvada Chugh, Ewa K. Paluch

**Affiliations:** 1 MRC Laboratory for Molecular Cell Biology, University College London, London WC1E 6BT, UK; 2 Institute for the Physics of Living Systems, University College London, London WC1E 6BT, UK; 3 Department of Physiology, Development and Neuroscience, University of Cambridge, Cambridge CB2 3DY, UK

**Keywords:** Actin, Cell shape, Cellular cortex, Contractility, Mechanics

## Abstract

Precisely controlled cell deformations are key to cell migration, division and tissue morphogenesis, and have been implicated in cell differentiation during development, as well as cancer progression. In animal cells, shape changes are primarily driven by the cellular cortex, a thin actomyosin network that lies directly underneath the plasma membrane. Myosin-generated forces create tension in the cortical network, and gradients in tension lead to cellular deformations. Recent studies have provided important insight into the molecular control of cortical tension by progressively unveiling cortex composition and organization. In this Cell Science at a Glance article and the accompanying poster, we review our current understanding of cortex composition and architecture. We then discuss how the microscopic properties of the cortex control cortical tension. While many open questions remain, it is now clear that cortical tension can be modulated through both cortex composition and organization, providing multiple levels of regulation for this key cellular property during cell and tissue morphogenesis.

## Abstract (precis)

**Summary:** A summary of the composition, architecture, mechanics and function of the cellular actin cortex, which determines the shape of animal cells, and, thus, provides the foundation for cell and tissue morphogenesis.

## Introduction

The cellular cortex is a thin actin network bound to the plasma membrane that is present in most animal cells. Cortical actin filaments are organized as a dense crosslinked meshwork containing over a hundred actin-binding proteins (ABPs), including myosin-2 motors. Myosin-2 pulls on actin filaments, generating contractile stresses in the network. These stresses give rise to cortical tension, a key determinant of cell surface tension. Gradients in cortical tension drive changes in shape, such as those observed during cell migration, cell division and tissue morphogenesis (Levayer and Lecuit, 2012; Maddox and Burridge, 2003; Maître et al., 2016; Matzke et al., 2001; Sedzinski et al., 2011; Stewart et al., 2011). Moreover, misregulation of cortex contractility has been linked to developmental defects, for instance in neural tube closure (Escuin et al., 2015), and diseases including cancer and immunodeficiency (Moulding et al., 2013; Remmerbach et al., 2009). In this article and the accompanying poster, we review the rapidly expanding literature about cortex composition and organization, and discuss how these affect cortical tension and, as a result, the function of the cortex in morphogenesis.

**Figure:**
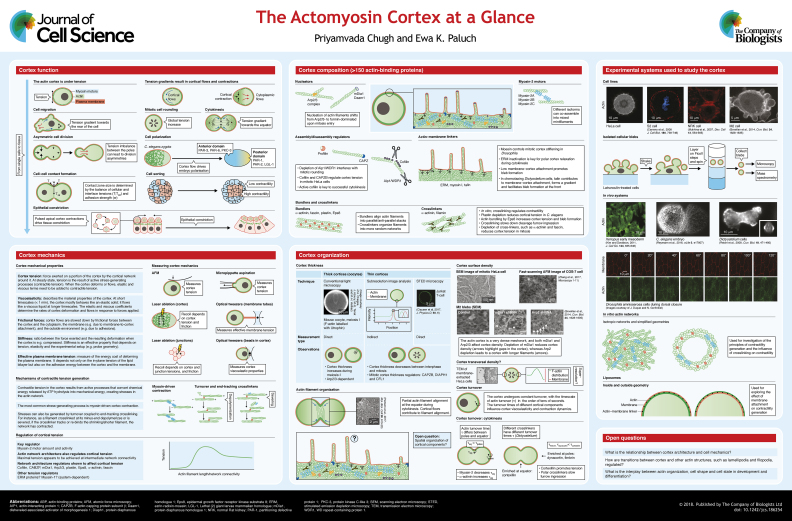

## Cortex function

The main function of the actin cortex is the control of animal cell morphogenesis. Local changes in cortex composition or organization can lead to cortical tension gradients, which result in local contractions and cellular deformations (see poster). For instance, during cell migration, cortical tension is usually higher at the back of the cell, powering cell body retraction (Chabaud et al., 2015; Vicente-Manzanares et al., 2009). Rearward contractility gradients also result in cortical flows throughout the cell body, which can be instrumental in generating the forces that propel migrating cells forward (Bergert et al., 2015; Lämmermann et al., 2008; Liu et al., 2015; Ruprecht et al., 2015). Cortex contractions can also result in the formation of blebs (see Box 1), which have been shown to function as leading-edge protrusions during cell migration in three-dimensional environments both in culture and *in vivo* (Blaser et al., 2006; Diz-Muñoz et al., 2016; Logue et al., 2015; Paluch and Raz, 2013; Zatulovskiy et al., 2014).

Box 1. Experimental systems used to study the cortex **Cell lines and cellular blebs**

HeLa cells (particularly in mitosis), *Drosophila* S2 cells, normal rat kidney cells and filamin-deficient melanoma M2 cells are the most common cultured cell lines used in cortex studies (see poster) (Carreno et al., 2008; Charras et al., 2006; Chugh et al., 2017; Kunda et al., 2008; Morone et al., 2006; Mukhina et al., 2007; Stewart et al., 2011).

Cellular blebs are also used as a model for the cortex (see poster). Blebs are spherical membrane protrusions driven by hydrostatic pressure generated in the cytoplasm by the contractile cortex (Cunningham et al., 1992). Blebs are initially devoid of cortex and re-assemble a cortical network *de novo* as they retract. Thus, they have been used as a convenient model system for the study of cortex assembly, particularly in M2 cells, which display constitutive prominent blebbing (Bovellan et al., 2014; Charras et al., 2006, 2008). Furthermore, blebs can be isolated, providing an enriched cortex fraction for proteomics (Biro et al., 2013).

***In vivo* systems**

*In vivo*, much of our understanding of the mechanisms controlling cortex contractions comes from studies in *Xenopus laevis*, *Dictyostelium discoideum*, *C.* *elegans* and *Drosophila* (see poster). *X. laevis* was one of the first systems where cortical instabilities were characterized (Capco et al., 1992), and continues to be used as a model for investigating contractions in development (Kim and Davidson, 2011). *Dictyostelium* cells are extensively used to study cortex dynamics, particularly during cell division (Reichl et al., 2008). In *C. elegans*, contractility-driven cortical flows have been well characterized during zygote polarization (Goehring et al., 2011; Mayer et al., 2010; Munro et al., 2004). *Drosophila* embryos are widely used to investigate apical cortex contractions during epithelial morphogenesis, for example, during ventral furrow formation, germ band extension and dorsal closure (Blanchard et al., 2010; Martin et al., 2009; Munjal et al., 2015; Solon et al., 2009).

***In vitro* systems**

Investigating the mechanisms of contractility generation in cells can be difficult because of redundancies between components and feedback loops interfering with specific perturbations. *In vitro* systems, using purified components in known concentrations, have been instrumental in expanding our understanding of contractility generation in cortex-like actomyosin networks.

*In vitro* studies have helped to formulate mechanisms for how myosin activity in isotropic cortical networks results in overall contractile forces (reviewed in Murrell et al., 2015). Recent work has also dissected the relationship between crosslinking, motor activity and network contractility (Alvarado et al., 2013; Ennomani et al., 2016). Finally, actomyosin contractility has been reconstituted at the surface of liposomes, allowing researchers to explore the effect of membrane attachment on contractility (Carvalho et al., 2013).

Precise modulation of cortex contractility also drives the series of shape changes underlying cell division (reviewed in Green et al., 2012; Ramkumar and Baum, 2016). Mitotic rounding displayed by cells in culture, as well as in tissues, is thought to be driven by reorganization of actin into a uniform cortical layer and a progressive increase in cortex tension (Cramer and Mitchison, 1997; Hoijman et al., 2015; Kondo and Hayashi, 2013; Stewart et al., 2011). Failure in mitotic rounding leads to defects in spindle assembly, pole splitting and a delay in mitotic progression (Lancaster et al., 2013). At the end of mitosis, a gradient in cortical tension from the poles towards the equator drives cleavage furrow ingression (Bray and White, 1988; Rappaport, 1967; Schwayer et al., 2016). Importantly, even though cell cleavage is driven by actomyosin accumulation in an equatorial contractile ring, a contractile cortex remains at the poles of the cell throughout cytokinesis (see poster). This polar cortex must be precisely controlled, as asymmetries in contractility between the two poles can lead to cell shape instabilities, aneuploidy and division failure (Sedzinski et al., 2011). Interestingly, a controlled asymmetry in polar contractility has been proposed to drive asymmetric division in neuroblasts (Cabernard et al., 2010; Connell et al., 2011; Ou et al., 2010; Tsankova et al., 2017).

Cortex tension can also contribute to cell polarization. In *Drosophila* neuroblasts, myosin-dependent asymmetric polar cortex extension during anaphase contributes to polarity protein segregation (Tsankova et al., 2017). Similarly, in one-cell *Caenorhabditis elegans* embryos, posterior-to-anterior actomyosin flows drive the segregation of PAR polarity proteins (Cheeks et al., 2004; Goehring et al., 2011; Mayer et al., 2010; Munro et al., 2004) (see Box 1).

During cell–cell contact formation, a local decrease in cortex tension at the cell–cell interface has been shown to control the size of the adhesion zone in zebrafish progenitor cells and mouse blastocysts (Maître et al., 2012, 2015; Manning et al., 2010). Experiments and modelling suggest that this decrease in interfacial cortex tension, much more than the changes in adhesion strength, control cell contact formation and as a result, cell sorting in tissues (Amack and Manning, 2012; Krieg et al., 2008; Maître et al., 2012).

Finally, in polarized epithelia, apical cortex contractions are often instrumental in tissue morphogenesis (reviewed in Coravos et al., 2017; Levayer and Lecuit, 2012). Interestingly, in many systems, overall tissue contractions have been shown to result from pulsed cortical contractions (Martin et al., 2009; Munjal et al., 2015; Solon et al., 2009). Pulsed contractions might facilitate overall tissue contraction by facilitating cell rearrangements (Curran et al., 2017). Oscillatory contractions could be an intrinsic property of the actomyosin cortex (Baird et al., 2017; Dierkes et al., 2014; Munjal et al., 2015, reviewed in Salbreux et al., 2012). However, in several systems, other processes, such as mechanical feedback loops involving adhesion molecules or an underlying oscillation in the activity of the small GTP-ase RhoA, have been proposed to stabilize cortex oscillations (Jurado et al., 2016; Nishikawa et al., 2017). The exact mechanism of pulsed cortex contractions might thus be cell type dependent.

## Cortex composition

A mass spectrometry study using isolated cellular blebs to obtain enriched cortical fractions identified over 150 ABPs in the cortex of constitutively blebbing human melanoma M2 cells (Biro et al., 2013) (see Box 1), and the function of specific cortical ABPs has been investigated in a number of other cell lines. We discuss here some cortex-specific functions of key ABPs (see poster).

### Actin filament nucleators

Cortical actin assembly appears to result from the combined action of formins, which nucleate and elongate linear actin filaments, and the Arp2/3 complex, which drives filament branch formation. Mass spectrometry combined with a visual screen has shown that in melanoma M2 cells, the cortex is nucleated predominantly by the formin mDia1 (encoded by *DIAPH1*) and the Arp2/3 complex (Bovellan et al., 2014). In mouse oocytes, the formin Daam1 also contributes to cortex assembly (Lu et al., 2017). Interestingly, in HeLa cells, mDia1, but not Arp2/3, is essential for cell division (Bovellan et al., 2014), and in *Drosophila* notum cells, cortical actin nucleation shifts from being dominated by Arp2/3 to formin upon mitosis entry (Rosa et al., 2015). Such shifts between nucleators could affect cortex architecture and mechanics.

### Regulators of actin assembly and disassembly

The most abundant regulators of actin assembly and disassembly that have been identified by mass spectrometry in M2 cell cortices are the capping proteins CAPZA and CAPZB, the actin-severing and -disassembling protein cofilin (CFL1 and CFL2), and profilin 1 (PFN1), which binds actin monomers and promotes actin polymerization (Biro et al., 2013). Precise regulation of cortical actin assembly is important during cell division. Depletion of either CFL1 or CAPZB increases cortex thickness and decreases cortex tension in mitotic HeLa cells, suggesting that regulation of actin filament length is crucial to tension control (Chugh et al., 2017, and discussion of cortex tension below). Depletion of the actin severing protein WDR1 (the human homologue of the yeast actin-interacting protein 1, Aip1) interferes with mitotic rounding, further suggesting defects in the build-up of cortical tension when F-actin assembly is perturbed (Fujibuchi et al., 2005).

The role of cofilin and its close relative actin-depolymerizing factor (ADF, also known as destrin) in mitosis has been controversial. In HeLa cells, ADF/cofilin has been reported to be inactivated in mitosis through phosphorylation by LIM kinase-1 (LIMK1) (Amano et al., 2002) and Aurora A (Ritchey and Chakrabarti, 2014), and this inactivation has been proposed to control cortex stability and stable spindle positioning (Kaji et al., 2008). However, CFL1 depletion affects the cortex in mitotic HeLa cells (Chugh et al., 2017), suggesting that cofilin maintains cortical activity in mitosis. It has also been proposed that phosphorylated ADF/cofilin some actin-severing activity (Fujibuchi et al., 2005).

During cytokinesis, active dephosphorylated cofilin is essential for successful cell division, possibly because it prevents an excessive F-actin accumulation during cleavage furrow formation (Kaji et al., 2003; Yang et al., 2004). Interestingly, in *Dictyostelium* cells, Aip1 depletion also leads to excessive cortical actin accumulation and cytokinesis failure (Ishikawa-Ankerhold et al., 2010). ADF/cofilin depletion results in the formation of abnormally large polar blebs, which appear to destabilize the spindle and lead to cytokinetic defects (Wiggan et al., 2012). Abnormal cortex contractions and spindle instabilities also occur upon depletion of profilin (*chickadee*) in *Drosophila* S2 cells (Dean et al., 2005), while in the *C. elegans* early embryo, profilin depletion impairs cortical actin assembly and leads to cytokinesis failure (Severson et al., 2002).

### Actin crosslinkers

Many different bundlers and crosslinkers have been found in the actin cortex, including α-actinin proteins, fascin, filamin proteins, plastin proteins (also known as fimbrin proteins) and Eps8 (Aoki et al., 2016; Biro et al., 2013; Charras et al., 2006; Logue et al., 2015; Luo et al., 2013; Werner et al., 2013). While bundlers (e.g. fascin and plastin) tend to align actin filaments into parallel and/or anti-parallel stacks, crosslinkers (e.g. filamin) organize them into more random networks. Interestingly, some proteins, such as α-actinin, appear to be able to promote both types of organization (Falzone et al., 2012). *In vitro* studies indicate that the level of crosslinking in the cortex is a key regulator of actomyosin contractility (Alvarado et al., 2013; Ennomani et al., 2016; Koenderink and Paluch, 2018). However, redundancies between crosslinkers may make it difficult to discern the functional role of crosslinking in the cellular cortex. Nonetheless, cortical tension is strongly reduced upon plastin deletion in *C. elegans* zygotes, leading to defects in polarization and cytokinesis (Ding et al., 2017). Extracellular signal-regulated kinase (ERK)-activated actin filament bundling by Eps8 has also been shown to increase tension and favour bleb formation during confined migration of cancer cells (Logue et al., 2015). Furthermore, a recent screen for regulators of mitotic cell mechanics has shown that the crosslinkers α-actinin and fascin contribute to the control of mitotic rounding force, which is related to cortical tension (Toyoda et al., 2017).

Crosslinkers also regulate cortex contraction dynamics; for example, increased crosslinking slows down cleavage furrow ingression in cytokinesis (Mukhina et al., 2007; Reichl et al., 2008). Interestingly, during *Dictyostelium* cytokinesis, distinct crosslinkers localize to the equator and the poles, suggesting that crosslinkers contribute to the local control of actin architecture during cell division (Reichl et al., 2008).

### Actin-membrane linkers

Cortical F-actin is tethered to the plasma membrane through actin-membrane linkers, including ezrin-radixin-moesin (ERM) proteins and myosin-1 motors (Biro et al., 2013; Bretscher et al., 2002). In *Drosophila* S2 cells, activation of moesin (the only ERM protein in *Drosophila*) is required for mitotic cortex stiffening and cell rounding, and as a result for spindle stability (Carreno et al., 2008; Kunda et al., 2008). In mammalian cells, ERMs are also activated in mitotic cells; their impaired activation does not interfere with rounding, but is required for proper spindle orientation (Machicoane et al., 2014). Furthermore, ERM inactivation at the cell poles appears to be crucial for relaxation of polar tension during cytokinesis (Kunda et al., 2012; Rodrigues et al., 2015; Roubinet et al., 2011).

Membrane-to-cortex attachment is also a key regulator of bleb-based cell migration, as blebs form at locations of reduced attachment (Paluch and Raz, 2013). Reduced ERM activity has been shown to be involved in bleb-based migration of cultured Walker carcinosaroma cells and zebrafish primordial germ cells (Goudarzi et al., 2012; Rossy et al., 2007), and interfering with either ERM or myosin-1B activity enhances bleb formation in migrating zebrafish progenitors during gastrulation (Diz-Muñoz et al., 2010). In chemotaxing *Dictyostelium* cells, talin contributes to attachment of the cortex to the membrane and forms a gradient towards the cell rear, facilitating bleb formation at the front (Collier et al., 2017).

### Myosin motors

Myosin-2 motors are a key cortex component, as they generate most of the cortical contractile tension (see below). A myosin-2 motor is a hexamer composed of two heavy chains, two regulatory light chains and two essential light chains. Non-muscle myosin-2 assembles into bipolar minifilaments that exert contractile forces in the cortex (Henson et al., 2017). All three myosin-2 heavy chain isoforms, MYH9 (myosin-2A), MYH10 (myosin-2B) and MYH14 (myosin-2C), have been shown to localize to the cortex (Beach et al., 2014; Biro et al., 2013; Maliga et al., 2013). The different isoforms can co-assemble into mixed minifilaments (Beach et al., 2014; Shutova et al., 2014). Whether specific isoforms exert distinct functions in the cortex remains poorly understood. Besides myosin-2, several myosin-1 isoforms, which are involved in membrane-to-cortex attachment, and myosin-18, which can co-assemble with myosin-2 (Billington et al., 2015), also localize to the cortex (Maliga et al., 2013).

In addition to the proteins discussed here, a number of signalling pathways affect cortex organization and mechanics, and have been extensively reviewed elsewhere (Heng and Koh, 2010; Zaidel-Bar et al., 2015).

## Cortex organization

The mechanical properties of the cortex are governed not only by its composition but also by how the components are spatially organized. Cortex architecture remains poorly understood owing to the sub-resolution dimensions of the cortex. However, recent studies have started unveiling the nanoscale organization of the cortex (see poster).

### Cortex thickness

In some large oocytes, cortex thickness can be directly measured via optical microscopy. For instance, in meiotic mouse oocytes, an Arp2/3-dependent cortex thickening from ∼1.4 to 4 µm has been reported (Chaigne et al., 2013). Interestingly, this thickening is associated with the exclusion of myosin-2 from the cortex and a decrease in cortex tension (Chaigne et al., 2013). In smaller cells, cortex thickness is usually below the resolution of conventional light microscopy. Transmission electron microscopy (TEM) in *Dictyostelium* cells and in retracting blebs in melanoma M2 cells suggests that the cortical layer spans ∼100 nm (Charras et al., 2006; Hanakam et al., 1996). More recently, sub- and super-resolution techniques have been used to assess cortex thickness via light microscopy. Clark et al. have used sub-resolution analysis of confocal images to extract cortical thickness from the relative localization of cortical actin and the plasma membrane, revealing thicknesses of ∼200 nm in mitotic HeLa cells (Clark et al., 2013). Clausen et al. directly measured an approximate cortex thicknesses of 130 nm in Jurkat cells by performing stimulated emission depletion (STED) microscopy and also reported the existence of a gap (∼20 nm) between the cortex and the membrane (Clausen et al., 2017). Interestingly, cortical thickness appears to be precisely controlled, as the thickness of the cortex that reassembles *de novo* in a bleb sometimes overshoots, but then returns to original values as the bleb retracts (Clark et al., 2013). We recently reported that cortical thickness in mitotic HeLa cells is primarily regulated by *CAPZB*, *DIAPH1* and *CFL1*, which control actin assembly and disassembly, and thus filament length (Chugh et al., 2017).

### Cortex density

Cortex density is likely to not only affect cortex mechanics, but also cortex–membrane interactions and diffusion of organelles through the cortical mesh. Scanning electron microscopy (SEM) and atomic force microscopy (AFM) have both been used to map the surface of the cortex, revealing typical mesh sizes of 100 nm or less (Charras et al., 2008; Chugh et al., 2017; Eghiaian et al., 2015; Kronlage et al., 2015) (see poster). Cortical mesh size appears to be affected by actin nucleators (Bovellan et al., 2014; Eghiaian et al., 2015), and can also be modulated by drugs affecting actin polymerization dynamics (Kronlage et al., 2015; Zhang et al., 2017).

Notably, these techniques only probe the mesh size of the outermost layer of the cortex. Electron tomography of ∼100-nm-thick sub-membrane layers obtained by shearing off the top surface of the cell has given some insight into cortex organization on the cytoplasmic side, and revealed mesh sizes between 50 and 200 nm, depending on the cell type (Morone et al., 2006). How the cortex is organized in the transverse direction remains largely unknown, although a recent super-resolution study suggests that there is a gradient in the transversal density of the cortex, with a maximum actin density at a finite distance from the membrane (Clausen et al., 2017).

### Filament organization

Cortex mechanics is also likely to be affected by the length, polarity and spatial organization of cortical actin filaments. While little is known about filament polarity and length (Fritzsche et al., 2016), filament organization has been assessed in several systems. In rounded cells, cortical actin appears to be mostly isotropic (Bovellan et al., 2014; Morone et al., 2006). In contrast, partial filament alignment along the equator has been observed during cytokinesis using SEM and fluorescence-detected linear dichroism microscopy (Fishkind and Wang, 1993; Maupin and Pollard, 1986), and, more recently, super-resolution imaging (Fenix et al., 2016). In *C. elegans* zygotes, where the cortex is less dense than in cultured cells, equatorial filament alignment could be visualized in live cells and was shown to directly result from cortical flows towards the equator (Reymann et al., 2016). A recent study in sea urchin embryos showed, via super-resolution microscopy and TEM, that actin and myosin form contractile units in the cytokinetic ring, with anti-parallel actin filaments connected by regularly spaced myosin-2 minifilaments (Henson et al., 2017) (see poster).

### Cortex turnover

Finally, the relative turnover rates of cortex constituents are likely to have a strong influence on cortex viscoelasticity and contraction dynamics (Hiraiwa and Salbreux, 2016; Salbreux et al., 2012; Turlier et al., 2014). Cortical actin typically turns over within a few tens of seconds (Fritzsche et al., 2013; Mukhina et al., 2007). During cytokinesis, myosin-2 activity appears to accelerate actin turnover at the contractile ring (Guha et al., 2005; Murthy and Wadsworth, 2005), while crosslinking by α-actinin slows it down (Mukhina et al., 2007). In *Dictyostelium* cells, turnover of cortical components appears to be overall slower at the equator than at the poles (Srivastava and Robinson, 2015). Interestingly, in that system, while some crosslinkers (with fast turnover dynamics) slowed down furrow ingression, others such as cortexillin (with slow turnover dynamics) appeared to be instrumental for generating actomyosin tension (Reichl et al., 2008). The exact influence of turnover of cortex constituents on cortex mechanics remains poorly understood.

## Cortex mechanics

The mechanical property of the cortex that is key for its function in cell morphogenesis is cortical tension (see Box 2). Cortical tension is most prominently controlled by myosin-2 activity (Clark et al., 2014; Murrell et al., 2015; Salbreux et al., 2012). In addition, actin turnover could contribute to contractile tension generation. In order for turnover to generate tension, the polymerizing or depolymerizing filament ends must remain attached to the rest of the network either via crosslinkers that remain bound to a filament tip undergoing turnover, or other end-tracking proteins such as formins (see poster, reviewed in Clark et al., 2014). To what extent turnover-driven processes contribute to cortical tension has not been systematically investigated. However, inhibition of myosin activity can reduce cortical tension by as much as 80% (Chugh et al., 2017; Ramanathan et al., 2015), and estimates based on typical myosin-2-exerted forces indicate that myosin activity is sufficient to account for tension values that have been measured experimentally (Salbreux et al., 2012).

Box 2. Methods to measure cortex mechanics The mechanical property of the cortex most extensively investigated is tension. Absolute cortex tension measurements rely on direct cell micromanipulation, most commonly by AFM or micropipette aspiration (MPA) (see poster). In AFM measurements, tension is determined from the response of the cell to compression. The most-accurate AFM surface tension measurements are obtained with spherical cells compressed by a flat cantilever (Fischer-Friedrich et al., 2015; Haase and Pelling, 2015); this method is thus mainly applicable to rounded cells in isolation. AFM measurements have, for instance, shown that cortex tension increases between interphase and mitosis in cultured cells (Chugh et al., 2017; Stewart et al., 2011), that higher cortex tension promotes confined migration of cancer cells (Logue et al., 2015) and that tensile forces contribute to cell sorting during zebrafish gastrulation (Krieg et al., 2008). Recently, AFM measurements of mitotic rounding force, a readout of cortical tension, have been used to conduct a large-scale screen of regulators of cortex mechanics (Toyoda et al., 2017). In MPA measurements, tension is determined from the pressure required to aspirate a cell into a micropipette (Brugués et al., 2010; Evans and Yeung, 1989; Hochmuth, 2000). MPA only requires the portion of the cell near the pipette to be spherical and can thus be applied to cells adhering to a substrate. MPA tension measurements have shown that a threshold tension is required for cellular bleb formation (Tinevez et al., 2009), that cortical tension decreases in meiosis in mouse oocytes (Chaigne et al., 2013; Larson et al., 2010) and that tension decreases during post-ovulation ageing of mouse eggs (Mackenzie et al., 2016). Both AFM and MPA have been used extensively for the investigation of cortical tension regulation (Cartagena-Rivera et al., 2016; Chugh et al., 2017; Logue et al., 2015; Matthews et al., 2012; Ramanathan et al., 2015; Stewart et al., 2011; Tinevez et al., 2009; Toyoda et al., 2017).

An alternative method to assess cortical tension, which does not require direct contact with the cell and can thus be applied to cells in tissues, is laser ablation (see poster). Typically, a pulsed laser is used to locally disrupt the cortex and the resulting recoil of the network around the cut is measured (Mayer et al., 2012). This method has been extensively used to probe cortex mechanics in *C. elegans* and zebrafish embryos (Behrndt et al., 2012; Mayer et al., 2010; Saha et al., 2016). Laser ablations of cell–cell junctions or of entire cells are also commonly used to probe the mechanics of the apical cortex in epithelial tissues in developing embryos, particularly in *Drosophila* (Farhadifar et al., 2007; Kasza et al., 2014; Martin et al., 2010; Rauzi et al., 2010; Tetley et al., 2016), but also in *C. elegans* (Roh-Johnson et al., 2012) and mouse (Heller et al., 2014). Importantly, laser ablation experiments only yield relative cortex tension values, as both cortical tensile forces and frictional forces slowing down cortical flows influence recoil velocities.

Finally, optical tweezers have been used to probe cortical viscoelastic properties (reviewed in Hoffman and Crocker, 2009) and more recently, the mechanics of cell–cell junctions in *Drosophila* epithelia (Bambardekar et al., 2015).

The function and regulation of myosin-2 in controlling cortical tension has been extensively studied (Munjal et al., 2015; Ramanathan et al., 2015; Stewart et al., 2011; Tinevez et al., 2009). For a long time, the contribution of other aspects of the cortical network to tension has been largely overlooked, and myosin-2 reporter intensity is commonly used as a readout of tension in morphogenesis studies (Bergert et al., 2015; Chanet et al., 2017; Maître et al., 2016; Mayer et al., 2010; Ramanathan et al., 2015). However, since tension results from myosins exerting forces on the actin network, it is to be expected that, at a given myosin activity, network architecture would also influence tension. Indeed, several *in vitro* studies of reconstituted actomyosin networks showed that actin filament organization and crosslinking level strongly affect tension (Alvarado et al., 2013; Ennomani et al., 2016; Reymann et al., 2012). Recent studies make similar observations in cells (Chugh et al., 2017; Ding et al., 2017). Taken together, the emerging view is that cortical tension is maximal for an intermediate level of connectivity in the actomyosin network. Connectivity can be varied by diverse mechanisms, including by changes in the level of crosslinking (Ding et al., 2017), branching (Ennomani et al., 2016) or actin filament length (Chugh et al., 2017). When connectivity is too low, stresses cannot propagate within the network, and global tension is low. When connectivity is too high, the network is too rigid to remodel, and tension in the network is low. At optimum connectivity levels, the network is thought to be sufficiently connected for tension propagation and sufficiently malleable to allow for remodelling, favouring contractile configurations. The microscopic mechanisms that drive the observed tension maximum remain to be investigated (Chugh et al., 2017; Ennomani et al., 2016; reviewed in Koenderink and Paluch, 2018).

Proteins linking the actin cortex to the plasma membrane, such as ERM proteins and myosin-1 motors, have been shown to contribute to cortical tension in *Dictyostelium* (Dai et al., 1999) and mouse oocytes (Larson et al., 2010). However, in other cell types, membrane-to-cortex attachment does not appear to affect cortical tension (Diz-Muñoz et al., 2010), but rather controls effective plasma membrane tension, which is a measure of how easy it is to deform the membrane, for example, during protrusion formation (Diz-Muñoz et al., 2010; Nambiar et al., 2009). In *Dictyostelium* cells, membrane-to-cortex attachment via moesin has also been shown to control cortical stiffness and to be required for mitotic rounding (Kunda et al., 2008). Importantly, cortical stiffness is distinct from cortical tension: stiffness, which is a measure of the ratio between the forces applied and the extent of the resulting deformation, is an effective property that depends on the experimental configuration, whereas cortical tension is a property of the cortex at any given time, and does not depend on how it is measured (for definitions, see poster and Salbreux et al. 2012). Stiffness generally depends on cortical tension, but could also be influenced by other physical parameters. These two properties could thus be controlled by different mechanisms (Cartagena-Rivera et al., 2016; Harris et al., 2012).

Finally, membrane-to-cortex attachment could also affect cortex contractions by generating a friction that slows down cortex flows (Mayer et al., 2010; Murrell et al., 2015). The dynamics of cortex contractions and the length scales over which they propagate also depend on the level of crosslinking of the cortex and on the rate of turnover of these crosslinks, which determine the viscoelastic material properties of the cortex (Murrell et al., 2015; Reichl et al., 2008) (see Box 2).

## Conclusions and perspectives

Contractility of a ‘superficial plasma gel layer’ at the cell surface was proposed to be a key driver of cell shape changes at the end of the 19th century (discussed in Rappaport, 1996), and parallels between cleavage furrow ingression and cell rear contraction during migration had already been discussed as early as 1939 (Lewis, 1939). However, for a long time, our comprehension of cortex contractility in non-muscle cells remained very limited (discussed in Bray and White, 1988). The last 10 years have brought a remarkable leap in our understanding of the cellular actin cortex. Both cortex composition and its mechanical properties are now relatively well understood, and how changes in composition and mechanics drive specific shape changes is beginning to be unveiled.

The biggest challenge for the coming years will be to truly bridge the molecular and cellular scales in cortex studies. Recent work has highlighted that the nanoscale organization of the cortex, in addition to the concentration and activities of its components, is key to the control of cortical tension. While some methods are available to probe the surface architecture of the cortex, how the network is organized in the transverse direction remains essentially a black box. New tools, possibly combining super-resolution and electron microscopy, will be required to unveil the transversal density, orientation and polarity of cortical actin filaments, as well as the localization of other cortex components involved in tension generation.

Another open question is how the cortex is being remodelled when it transitions into other actin structures, such as lamellipodia during cell spreading. Lamellipodia, stress fibres and the cortex appear to contain essentially the same molecular components. It is currently unclear what determines which network structure dominates at any given time. Studies of cell shape changes in development and differentiation, where transitions between cell states are often associated with transitions in cell shape and actin organization (Chalut and Paluch, 2016), are likely to unveil some key determinants of actin network architecture. These are likely to be driven by intracellular pathways, as well as environmental signals. As our understanding of the cortex in isolation matures, the interplay between cell state, the extracellular environment, cell shape and intracellular actin organization are an exciting and vastly unexplored research avenue.
